# Arthrospira Enhances Seroclearance in Patients with Chronic Hepatitis B Receiving Nucleos(t)ide Analogue through Modulation of TNF-α/IFN-γ Profile

**DOI:** 10.3390/nu14142790

**Published:** 2022-07-06

**Authors:** Sheng-Jie Shiue, Chao-Ling Cheng, Han-Shiang Shiue, Chun-Nan Chen, Sheng-Wei Cheng, Li-Wei Wu, Ganbolor Jargalsaikhan, Tze-Sian Chan, Hsin-Yi Lin, Ming-Shun Wu

**Affiliations:** 1 Division of Gastroenterology, Department of Internal Medicine, Wan Fang Hospital, Taipei Medical University, Taipei 116, Taiwan; sjshiue@tmu.edu.tw (S.-J.S.); 97431@w.tmu.edu.tw (C.-L.C.); hailey4824@gmail.com (H.-S.S.); 86662@w.tmu.edu.tw (C.-N.C.); 97427@w.tmu.edu.tw (S.-W.C.); tzesian@w.tmu.edu.tw (T.-S.C.); 2 Integrative Therapy Center for Gastroenterologic Cancers, Wan Fang Hospital, Taipei Medical University, Taipei 116, Taiwan; 3 Division of Gastroenterology, Department of Internal Medicine, Taiwan Adventist Hospital, Taipei 105, Taiwan; 4 Department of Internal Medicine, National Taiwan University Hospital, YunLin Branch, YunLin 640, Taiwan; b8501109@gmail.com; 5 Liver Center, Ulaanbaatar 14230, Mongolia; ganbolor.j@gmail.com; 6 Division of Gastroenterology and Hepatology, Department of Internal Medicine, School of Medicine, College of Medicine, Taipei Medical University, Taipei 110, Taiwan; 7 Institute of Chemical Engineering, National Taipei University of Technology, Taipei 106, Taiwan; 8 Institute of Biochemical and Biomedical Engineering, National Taipei University of Technology, Taipei 106, Taiwan; 9 International Ph.D. Program in Medicine, College of Medicine, Taipei Medical University, Taipei 110, Taiwan

**Keywords:** chronic hepatitis B, quantitative hepatitis B surface antigen, IFN-γ, B cell activation, NK cell activation

## Abstract

Chronic hepatitis B (CHB) virus infection, causing immune dysfunction and chronic hepatitis, is one of the leading risk factors for hepatocellular cancer. We investigated how Arthrospira affected hepatitis B surface antigen (HBsAg) reduction in CHB patients under continued nucleos(t)ide analogues (NA). Sixty CHB patients who had been receiving NA for at least one year with undetectable HBV DNA were randomized into three groups: control and oral Arthrospira at 3 or 6 g daily add-on therapy groups. Patients were followed up for 6 months. Oral Arthrospira-diet mice were established to investigate the possible immunological mechanism of Arthrospira against HBV. Within 6 months, mean quantitative HBsAg (qHBsAg) decreased in the oral Arthrospira add-on therapy group. Interestingly, interferon gamma (IFN-γ) increased but TNF-α, interleukin 6 (IL-6), hepatic fibrosis, and steatosis decreased in the add-on groups. In mice, Arthrospira enhanced both innate and adaptive immune system, especially natural killer (NK) cell cytotoxicity, B cell activation, and the interleukin 2 (IL-2), IFN-γ immune response. Arthrospira may modulate IL-2- and TNF-α/IFN-γ-mediated B and T cell activation to reduce HBsAg. Also, Arthrospira has the potential to restore immune tolerance and enhance HBsAg seroclearance in CHB patients through promoting T, B, and NK cell activation.

## 1. Introduction

Hepatitis B virus (HBV) infection is one of the most serious infectious diseases; indeed, more than 240 million people in the world become infected in their lifetime [1,2]. Chronic hepatitis B (CHB) patients have an elevated serum HBV DNA concentration, which is the main risk factor for liver fibrosis. Repeated liver fibrosis evolves to cirrhosis with symptomatic complications such as jaundice, ascites, variceal hemorrhage, and hepatic encephalopathy, and finally to hepatocellular carcinoma (HCC) [3].

The progression of CHB can be diagnosed by serological markers. Hepatitis B surface antigen (HBsAg) is one of the most accurate markers for CHB due to its earlier appearance and longer persistence in serum during HBV infection. The goals of the HBV treatment are loss of HBV DNA; loss of Hepatitis B virus e antigen (HBeAg) and acquisition of anti-HBeAg antibodies; improved histology; HBsAg-seroconversion; loss of HBsAg; and formation of anti-HBs antibodies [4]. Currently, two treatment options are available for chronic HBV infection: nucleoside/nucleotide analogues (NAs) or PEGylated interferon (PEG IFN) [5,6]. Due to a high relapsing rate after cessation of NAs, indefinite treatment until HBsAg seroclearance or even seroconversion to anti-HBs is recommended [3,5,7]. CHB has been treated for decades using interferon therapy [8,9]. However, interferon has been limited in its use due to its high rate of dose-related adverse effects [10]. HBsAg loss is uncommon due to the existence of covalently closed circular DNA (cccDNA) in the nucleus. HBsAg loss occurs in approximately 3–5% of PEG-IFN-treated patients and 0–3% of NA-treated patients [11]. Thus, searching for a novel solution that helps to eliminate HBsAg will be a great advance in CHB treatment.

Arthrospira species is a cyanobacterium that has been used frequently as a dietary supplement since ancient times and is known for its marked amount of proteins, phycobiliproteins, γ-linolenic acid, vitamins, and minerals [12,13]. Based on high-performance liquid chromatography (HPLC) analysis, Arthrospira contains high concentrations and good ratios of several key minerals for biosynthesizing β-carotene and ascorbic acid, and has the ability to maintain natural killer (NK) cell growth and to enhance B cell maturation [14,15]. As for the immune modulation of Arthrospira, it also enhances the activation of immune cells such as macrophages and T cells [14,16,17]. In addition, the anti-inflammatory effect of Arthrospira extract is at least partly responsible for the induction of an endotoxin tolerance-like state in macrophages [18]. Phycocyanin is one of the major phycobiliproteins in Arthrospira; it inhibits the levels of epithelial mesenchymal transition (EMT) [19], connective tissue growth factor (CTGF), and α-smooth muscle actins (α-SMA) [20], which play central roles and are clinically relevant in HBV-induced hepatic fibrosis [21,22,23]. All the reports have suggested that Arthrospira has the potential to modulate immune response against HBV, maybe enhance the IFN immune response, and reduce hepatic fibrosis.

In this randomized trial study, we investigated the role of giving Arthrospira to CHB patients who had been treated with NAs for at least one year and had achieved undetectable HBV DNA. The primary end point of this study was to evaluate the changes of quantitative HBsAg (qHBsAg) in three groups. The secondary end point was to investigate the immune response. We investigated the changes in cytokines, interferon gamma (IFN-γ), tumor necrosis factor-α (TNF-α), and interleukin 6 (IL-6) along with aspartate aminotransferase (AST), alanine aminotransferase (ALT) level, and hepatic fibrosis in three regimens. In the mice, we examined the effects of Arthrospira on B cell activation and immune response.

## 2. Materials and Methods

### 2.1. Study Population

**Figure 1:**
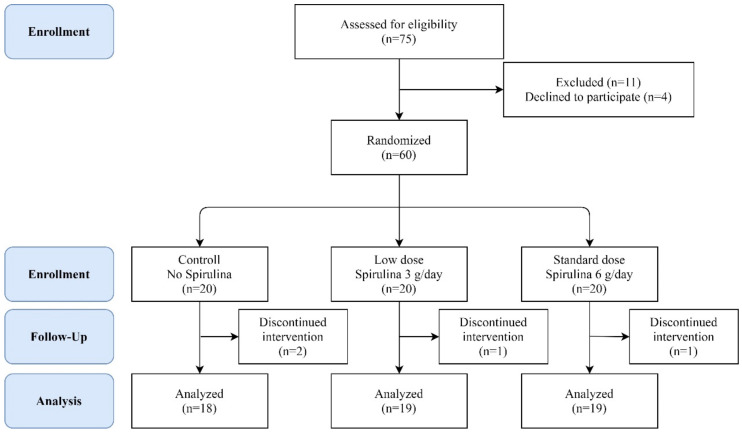
Flowchart for enrollment. Schemes follow another format. A total of 350 patients were enrolled into the initial evaluation of the study. Finally, 60 CHB patients were randomized into three groups: control and oral Arthrospira at 3 or 6 g daily add-on therapy groups. Four patients decided not to continue and left the trial earlier.

The study was conducted in the Taipei Medical University-Wan Fang Hospital in Taiwan. The intervention period was between December 2016 and January 2018. A total of 350 patients were recruited and assessed for eligibility to participate in the clinical trial. The age of patients ranged from 20 to 75 years old, each having undergone NA treatment for at least one year with undetectable HBV DNA (<20 IU/mL). None of these patients had undergone interferon therapy before this trial. A total of 290 patients that either did not meet the inclusion criteria or declined to participate were excluded. Finally, 60 CHB patients were enrolled and randomly allocated into three groups for the six-month study: the control group (only receiving NA), the low-dose group receiving Arthrospira (Far East Bio-Tec. Co., Ltd., Taiwan) at 3 g daily with NA, and the standard-dose group receiving Arthrospira at 6 g daily with NA (Figure 1). The levels of serum qHBsAg, IFN-γ, and IL-6 of CHB patients were measured at 0, 1, 3, and 6 months during the trial. The levels of liver fibrosis were measured at 0, 3, and 6 months during the trial. The protocol was approved by the Joint committee of Institutional review board (J-IRB) of Taipei Medical University (N201608026) and registered at ClinicalTrials.gov (accessed on 27 October 2016) (NCT02953600).

### 2.2. Liver Stiffness and Steatosis Measurement

Hepatic fibrosis and steatosis were analyzed by non-invasive Fibroscan^®^ vibration-controlled transient elastography with controlled attenuation parameter (CAP) to evaluate the liver stiffness (kPa) and CAP (dB/m) as per our previous clinical trial [24]. Liver stiffness is an indicator of liver fibrosis and CAP is considered a surrogate marker for hepatic steatosis [25]. The liver stiffness measurement (LSM) was performed with non-invasive FibroScan (Echosens, Paris, France) with an M or XL-probe, in an overnight fasting state, by an experienced technician. The right lobe of the liver was measured through the intercostal space when patients lay in supine position with their right arms at maximal abduction. LSM resulted as a median value in kilopascals (kPa) from 10 successful measurements [26]. FibroScan LSM cut-offs for Metavir scores were 6, 9, and 12 kPa for moderate fibrosis, advanced fibrosis, and cirrhosis for CHB patients, respectively. CAP was expressed in decibels/meter (dB/m), ranging from 100 to 400 dB/m, with values indicating S0, S1, S2, and S3 (<215, 252, 296, and >296 dB/m) as without, mild, moderate, and severe steatosis, respectively.

### 2.3. ELISA Analysis

Blood samples from subjects were collected and allowed to clot for 2 h at room temperature. Sera were centrifugated at 2500 rpm for 10 min and frozen for measurement. Serological IL-6, IL-2, TNF-α, and IFN-γ measurements were performed using ELISA (R&D, Minneapolis, MN, USA) according to the manual. The levels of serum IL-6, IL-2, TNF-α, and IFN-γ of CHB patients were measured at 0, 1, 3, and 6 months during the trial.

### 2.4. Mice

Balb/c mice were purchased from BioLASCO (Taipei, Taiwan). All mice were hosted in specific pathogen-free conditions and were used at an age between 8 and 17 weeks. All animal experiments were approved by the Taipei Medical University Committee of Experimental Animal Care and Use (approval No. WAN-LAC-108-015), performed according to its guidelines and those of the Council of Agriculture Guidebook for the Care and Use of Laboratory Animals.

### 2.5. Mice Treatment and Serum Antibody Measurement

To test innate immunization response during Arthrospira treatment, twenty 8-week-old mice were fed with sterile water or different dosages of Arthrospira (615, 1230, 2460 mg/kg; Far East Bio-Tec. Co., Taipei, Taiwan) for 6 weeks. Sera samples were harvested at the beginning and 3 weeks during treatment. For adaptive immunization response, twenty mice were immunized with 50 and 100 μg of DNP-ovalbumin (OVA) in complete Freund’s adjuvant at 5 and 7 weeks, respectively. Sera were collected at 0, 3, 5, 6, 7, 8, and 9 weeks during treatment. Antibody titers of sera IgM and IgG were measured by ELISA Quatitation Set (Bethy, Taipei, Taiwan).

### 2.6. In vitro Stimulation for Innate Response and Adaptive Response

For innate response, splenocytes from mice with innate immunization treatment were stimulated by adding ConA (5 μg/mL) in medium. For adaptive response, splenocytes from OVA-immunized mice were stimulated with ConA (5 μg/mL) and OVA (0.1 mg/mL). After stimulation for 48 h, the culture supernatant was used for the measurement of IL-2 and IFN-γ using the ELISA kit (eBioscience, Thermo Fisher, Waltham, MA, USA).

### 2.7. Phagocytosis Assays for Innate Immunity

Whole blood was collected in EDTA tubes and assays were performed using the pHrodo™ red phagocytosis particle labeling kit for flow cytometry (Thermo Fisher, Waltham, MA, USA). Samples were stained with mouse anti-human CD14 AF647 (clone TUK4, 1:20; AbD Serotec, Kidlington, UK).

### 2.8. Measurement of NK Cell Cytotoxic Activity for Innate Immunity

YAC-1 target cells (2 × 10^6^ cells; Food Industry Research and Development Institute, Hsinchu, Taiwan, ROC) were labeled with carboxyfluorescein succinimidyl ester (CFSE; final concentration of 2 μM) to discriminate target cells from effector cells. The effector cells (NK cells) were isolated from splenocytes of mice with three different doses of Arthrospira (615, 1230, or 2460 mg/kg/day). Then, isolated NK cells were incubated with CFSE-labeled YAC-1 target cells at different effector-to-target (E:T) ratios of 6.25:1 and 12.5:1 in 96-well plates with 100 μL of RPMI 1640 medium, and 50,000 target cells were used constantly. The positive control with the NK92 cell line was tested with YAC-1 cells at an E:T ratio of 32:1, and a negative control with YAC-1 cells alone was also incubated in each test. After co-culture for 4 h at 37 °C and 5% CO_2_, the cell mixture was stained with 5 μL of 7-AAD (Beckman Coulter, Milan, Italy) for 15 min in the dark. Flowcytometry data were analyzed on CytoFLEX S Flow Cytometer (Beckman Coulter, Brea, CA, USA), using CytExpert software version 2.4 (Beckman Coulter). NK cytotoxicity (%) was calculated as cells positive for both CFSE and 7-AAD/total CFSE positive cells, after subtracting the spontaneous lysis (%) in the negative control [27,28].

### 2.9. Statistical Analysis

The data in Table 1 were analyzed with R version 3.2.5. The normalization of the results in Table 1 was processed using the Shapiro–Wilk test, and then the assumption of homogeneity for the residuals and variables was checked using the Bartlett test. Statistical comparisons for other data were performed using GraphPad Prism 9 software. All the data have been expressed as mean ± SEM; n indicates the number of animals in each group. Two-way analysis of variance (ANOVA) with *post hoc* Tukey test was used to compare the data of different groups. *p* < 0.05 was considered statistically significant.

## 3. Results

### 3.1. Baseline Data in the Randomized Double-Blind Clinical Trial

Demographic parameters, body mass index (BMI), and blood chemistry levels of the three groups of CHB patients are reported in Table 1. The study population was predominantly male, with 43 men and 17 women distributed evenly among the three groups, showing no significant differences among gender, age, BMI, qHBsAg, AST, ALT, or liver stiffness. Similar levels of other blood chemistry markers such as creatinine (CA), uric acid (UA), and vitamin B12 (B12) were measured among the groups. No significant difference of demographic parameters was found from the recruited CHB subjects after the randomized, double-blind allocation. Four patients dropped out of this study; a total of 18, 19, and 19 patients retained in the control, low-dose, and standard-dose groups, respectively (Figure 1).

### 3.2. Arthrospira Reduces Serum qHBsAg and qHBcrAg Levels in CHB Patients receiving NA Treatment

**Figure 2:**
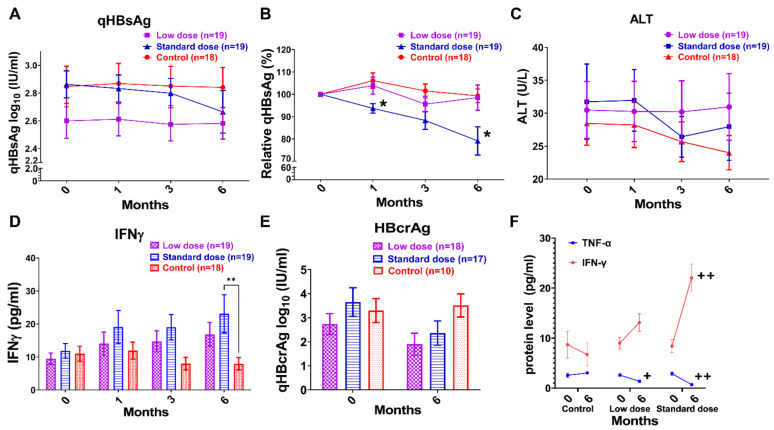
Arthrospira has the potential to reduce serum qHBsAg and qHBcrAg as well as to modulate serum TNF-α/IFN-γ profile in chronic hepatitis B virus infection patients receiving nucleos(t)ide analogue (NA) treatment. (**A**) The graph demonstrates the change of serum HBsAg quantity (qHBsAg) log_10_ (IU/mL) in patients in the three groups for 6 months. (**B**) The graph demonstrates the change from baseline of qHBsAg in patients in three groups for 6 months. The qHBsAg level at 0 months (before Arthrospira supplement) was as the baseline level of each patient in graph B. The reduction of qHBsAg and significant reduction of relative qHBsAg level were observed in the standard-dose group. (* *p* < 0.05, compared to control). (**C**) The ALT level (U/L) was reduced at 3 and 6 months both in the standard-dose and control groups. (**D**) Serum interferon gamma (IFN-γ) level (pg/mL) was increased in both Arthrospira add-on groups (** *p* < 0.01, compared to control). (**E**) The reduction of Hepatitis B core-related antigen (HBCrAg) quantity (qHBcrAg log_10_ IU/mL) was observed at 6 months in both Arthrospira add-on groups. (**F**) The graph demonstrates the TNF-α/IFN-γ profiles in patients in the three groups for 6 months. Serum IFN-γ level (pg/mL) was increased but serum TNF-α level (pg/mL) was decreased at 6 months in both Arthrospira add-on groups (+ *p* < 0.05 and ++ *p* < 0.01, compared to baseline level in each group). Control group: NA only; low-dose group: oral Arthrospira at 3 g daily with NA; standard-dose group: oral Arthrospira at 6 g daily with NA. Each data point represents mean ± SEM (%).

**Figure 3:**
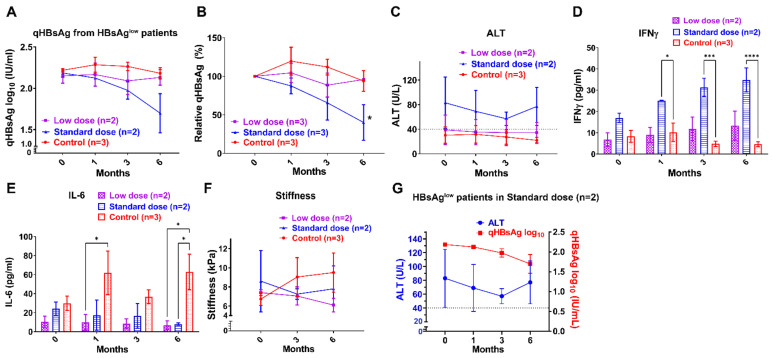
Arthrospira reduces serum qHBsAg and enhances serum IFN-γ level in qHBsAg^low^ patients receiving NA treatment. (**A**) The graph demonstrates the change in serum HBsAg quantity (qHBsAg) log_10_ (IU/mL) in patients in the three groups over 6 months. (**B**) The graph demonstrates the change from baseline of qHBsAg in patients in the three groups over 6 months. The qHBsAg level at 0 months (before Arthrospira supplement) was as the baseline level of each patient in graph B. A reduction in qHBsAg and significant reduction in relative qHBsAg levels were observed in the standard-dose group. (* *p* < 0.05, compared to control). qHBsAg^low^ patients:HBeAg(−) patients with qHBsAg 100–200 IU/mL. (**C**) The ALT level was increased above the upper limit of normal; 40 U/L in the standard does group. (**D**–**F**) Serum IFN-γ level was increased but serum IL-6 level and liver stiffness degree were decreased in both Arthrospira add-on groups. A significant increase in IFN-γ level was observed at 1, 3, and 6 months in the standard-dose group (* *p* < 0.5, *** *p* < 0.001 and **** *p* <0.0001, compared to control). Significantly lower IL-6 levels were observed at 6 months in both Arthrospira add-on groups (* *p* < 0.5, compared to control). (**G**) Combined HBsAg/ALT kinetics in patients in the standard-dose group (n = 4). The control group: NA only, n = 3; low-dose group: Arthrospira at 3 g daily with NA, n = 2; standard-dose group: Arthrospira at 6 g daily with NA, n = 2. Each data point represents mean ± SEM (%).

All participants in this study received follow-up examination at 1, 3, and 6 months during the trial. Figure 2A shows a steady but small qHBsAg decline over time in the standard dosage Arthrospira supplement; the qHBsAg log_10_ value was 2.86 ± 0.10 at 0 months, down to 2.66 ± 0.15 at 6 months. Interestingly, the qHBsAg levels decreased to below 100 IU/mL in three HBeAg-negative patients taking the standard-dosage Arthrospira supplements (722 IU/mL to 8 IU/mL; 172 IU/mL to 29 IU/mL; 136 IU/mL to 86 IU/mL). Previous studies demonstrated that qHBsAg levels < 100 IU/mL might provide a prediction of future HBsAg seroclearance [29,30]. After normalization with baseline, Figure 2B shows a significant and continually decreasing in serum qHBsAg at 6 months of standard-dose Arthrospira consumption (* *p* < 0.05, compared to control). For HBeAg-positive patients (n = 7, 4, and 2 in control, low-dose, and standard-dose groups, respectively), the qHBsAg log_10_ values were 3.19 ± 0.19 and 3.22 ± 0.37 at 0 months, down to 3.06 ± 0.15 and 3.19 ± 0.34 at 6 months in the low-dose and standard-dose groups, respectively (Supplementary Figure S1A). After normalization with baseline, Supplementary Figure S1B shows a significantly decrease the in low-dose group (100% down to 74%) and a smaller decrease in the standard-dose group (100% down to 94%) (* *p* < 0.05, compared to control). For HBeAg-negative patients (n = 11, 15, and 17 in control, low-dose, and standard-dose groups, respectively), the qHBsAg log_10_ value was 2.82 ± 0.10 at 0 months, down to 2.60 ± 0.16 at 6 months in the standard-dose Arthrospira group (Supplementary Figure S2A). After normalization with baseline, Supplementary Figure S2B shows a significant and continual decrease in the standard-dose group (100% down to 77%) (* *p* < 0.05, compared to control). For patients with qHBsAg^low^ (HBeAg(−), qHBsAg levels between 200 and 100 IU/mL; n = 3, 2, and 2 in control, low-dose, and standard-dose groups, respectively), the qHBsAg levels precipitously decreased in the standard-dose group; the qHBsAg log_10_ value was 2.18 ± 0.05 at 0 month, down to 1.69 ± 0.23 at 6 months (qHBsAg decline of −0.49 log_10_ IU/mL over 6 months, Figure 3A). After normalization with baseline, Figure 3B shows a significant and continual decrease in the standard-dose group (100% down to 40%, * *p* < 0.05, compared to control). This might predict future HBsAg seroclearance; Jeng’s study showed a steady qHBsAg decline over time in inactive carriers, and then the level of HBsAg precipitously declined (>0.5 log_10_ IU/mL in 1 year) before HBsAg loss [31]. All the data supported that Arthrospira has the potential to enhance the HBsAg decline in CHB patients receiving NA treatment. Although NAs strongly suppress HBV DNA, they do not affect cccDNA. Our recent ongoing clinical trial (NCT04718831) showed that Arthrospira could reduce hepatitis B core-related antigen (HBcrAg), which reflects clearance of cccDNA. In HBeAg-negative patients (n = 10, 18, and 17 in control, low-dose, and standard-dose groups, respectively), the quantity of HBcrAg log_10_ values were 2.73 ± 0.43 and 3.65 ± 0.59 at 0 months, down to 1.90 ± 0.45 and 2.36 ± 0.50 at 6 months in the low-dose and standard-dose groups, respectively (Figure 2E). However, the qHBcrAg log_10_ value was 3.30 ± 0.50 at 0 months, up to 3.51 ± 0.48 at 6 months in the control group (Figure 2E).

### 3.3. Arthrospira Enhances the Serum IFN-γ Level but Reduces Serum TNF-α in CHB Patients

Arthrospira has been found to modulate the activation of macrophages, NK cells, T cells, and B cells [16,17]. Since we detected the decrease of serum qHBsAg in subjects taking the higher dose of Arthrospira, we hypothesized that Arthrospira enhanced the IFN immune response and triggered T, B, and NK cell activation. We collected blood samples from each subject at each time point to determine the levels of serum cytokines with ELISA. As shown in Figure 2D, the IFN-γ expression increased in patients receiving the Arthrospira supplement, and significant increased IFN-γ levels were observed at 6 months in the standard-dose group (** *p* < 0.01, compared to control). Similar results were observed in HBeAg-positive, HBeAg-negative, and qHBsAg^low^ patients (Figure 3D, Supplementary Figures S1D and S2D). Interestingly, in subjects showing a significantly and continuously decreasing HBsAg level, significant increases in IFN-γ levels were observed (Figure 2B,D and Figure 3B,D; and Supplementary Figure S1B,D). Therefore, we suggest that Arthrospira has the potential to modulate the TNF-α/IFN-γ profile immune response in CHB patients. TNF-α/IFN-γ-expressing HBV-specific CD4 and CD8 T cells play critical roles in viral clearance and liver injury in CHB patients [32,33]. Our recent ongoing clinical trial (NCT04718831) showed that Arthrospira could modulate the TNF-α/IFN-γ profile in HBeAg-negative patients. In Figure 2F, significantly increasing IFN-γ level but decreasing TNF-α levels were observed at 6 months in patients receiving the Arthrospira supplement (n = 10, 18, and 17 in control, low-dose, and standard-dose groups, respectively; + *p* < 0.05 and ++ *p* < 0.01, compared to baseline), suggesting that the differentiation of TNF-α-expressing CD4 and CD8 T cells into IFN-γ-expressing CD4 and CD8 T cells may be enhanced by Arthrospira.

### 3.4. Arthrospira Reduces Hepatic Inflammation, Fibrosis, and Steatosis in CHB Patients receiving NA Treatment

**Figure 4:**
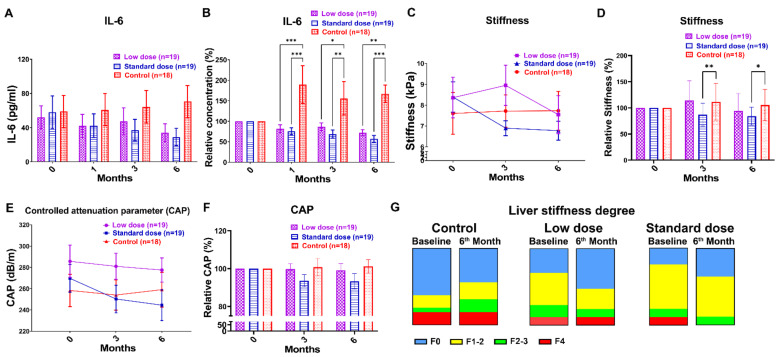
Arthrospira reduces interleukin 6 level, liver stiffness, and hepatic steatosis in CHB patients receiving NA treatment. (**A**,**C**,**E**) The graphs demonstrate the change in interleukin 6 (IL-6) level (pg/mL), liver stiffness (kPa), and controlled attenuation parameter (CAP, dB/m) in patients in the three groups over 6 months. (**B**,**D**,**F**) The graphs demonstrate the change from baseline of IL-6 level, liver stiffness degree, and CAP in patients in the three groups over 6 months. (**G**) The overall stiffness degree decreased in both groups with arthrospira supplementation but not in the control group. The bars show the proportion of liver stiffness degree in each group. F0: no fibrosis; F1–2: mild fibrosis; F2–3: severe fibrosis; F4: cirrhosis. The value at 0 month (before Arthrospira supplement) was as the baseline level of each patient in graphs B, D, and F. The IL-6, liver stiffness, and CAP were decreased at 6th month in both Arthrospira add-on groups. Significant reductions in relative IL-6 levels and stiffness were observed in the standard-dose group. (* *p* < 0.05, ** *p* < 0.01, *** *p* < 0.001, compared to control). Control group: NA only; low-dose group: oral Arthrospira at 3 g daily with NA; standard-dose group: oral Arthrospira at 6 g daily with NA. Each data point represents mean ± SEM (%).

HBV infection accounts for at least 50% cases of HCC worldwide [34]. Hepatic inflammation, steatosis, and cirrhosis also favor the process of carcinogenesis. Serum IL-6 levels have been shown to be closely related to HBV-associated liver cirrhosis and the risk of HBV-associated HCC [35]. Figure 4A shows that the IL-6 levels decreased in Arthrospira add-on groups but increased in the control group, suggesting that Arthrospira repressed the increase in IL-6 level in CHB patients. After normalization with baseline, Figure 4B shows a significant decrease in serum IL-6 level at 1, 3, and 6 months of low- and standard-dose Arthrospira consumption (* *p* < 0.05, ** *p* < 0.01, *** *p* < 0.001, compared to control). Similar results were observed in HBeAg-postive, HBeAg-negative, and qHBsAg^low^ patients (Figures S1E, S2E and Figure 3E, respectively); the IL-6 levels decreased in the Arthrospira add-on groups but increased in the control group. Liver stiffness is an indicator of liver fibrosis [25]. After Arthrospira supplementation for 6 months, liver stiffness was 8.35 ± 0.97 and 8.38 ± 0.72 at 0 months, down to 7.54 ± 0.90 and 6.77 ± 0.45 at 6 months in the standard- and low-dose groups, respectively, but was 7.60 ± 1.00 at 0 months, up to 7.72 ± 0.93 at 6 months in the control group (Figure 4C). After normalization with baseline, Figure 4D shows a significant decrease in liver stiffness degree at 3 and 6 months of standard-dose Arthrospira consumption (* *p* < 0.05 and ** *p* < 0.01, compared to control). Figure 4G shows that the overall stiffness degree decreased in both groups with Arthrospira supplementation but increased in the control group during therapy. Similar results were observed in HBeAg-negative and qHBsAglow patients (Figure 3F and Supplementary Figure S2F), the liver stiffness degrees decreased in the Arthrospira add-on groups but increased in the control group. For HBeAg-positive patients, liver stiffness was 6.44 ± 1.14, 10.05 ± 3.74, and 7.45 ± 0.65 at 0 months, down to 6.04 ± 0.91, 8.87 ± 2.77, and 5.85 ± 0.85 at 6 months in the control, low-dose, and standard-dose groups, respectively (Supplementary Figure S1F). CAP is an indicator for hepatic steatosis [25]. Figure 4E shows that the CAP values decreased in the Arthrospira add-on groups but increased in the control group at 6 months. After normalization with baseline, Figure 4F shows a decrease in liver CAP degree at 3 and 6 months of standard-dose Arthrospira consumption, with a smaller CAP decrease at 6 months in the low-dose group. All the data suggest that Arthrospira has the potential to reduce liver fibrosis and hepatic steatosis in CHB patients. Since all the data indicates that Arthrospira attenuates hepatic inflammation, hepatic steatosis, and cirrhosis in patients with CHB, Arthrospira has the potential to reduce the risk of HCC development in CHB patients [36].

### 3.5. Arthrospira Has the Potential to Trigger B Cell Activation

**Figure 5:**
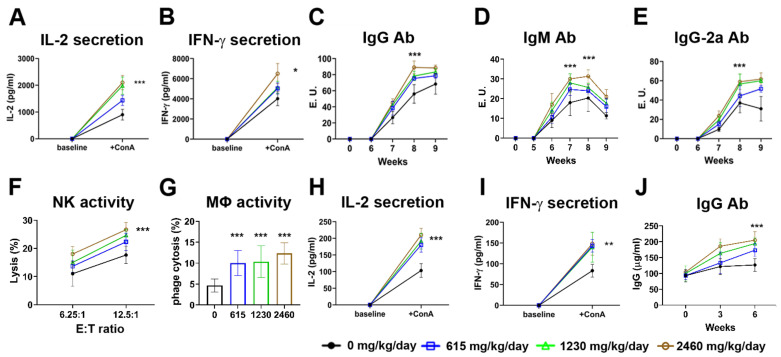
Arthrospira may trigger adaptive and innate immunities in vivo. (**A**,**B**) Arthrospira enhances IL-2 and IFNγ expression in ovalbumin (OVA)-immunized Balb/c mice. Splenocytes from OVA-immunized Balb/c mice were stimulated by adding ConA (5 μg/mL) in medium. Cytokine levels of IL-2 (pg/mL) and IFNγ (pg/mL) were detected using the ELISA kit. Significant increases in IL-2 and IFN-γ levels were observed in the Arthrospira 2460 mg/kg/day group (* *p* < 0.05 and *** *p* < 0.001, compared to control). (**C**–**E**) Arthrospira enhances IgG, IgM, and IgG 2a Ab level in OVA-immunized Balb/c mice. Balb/c mice with three different doses of Arthrospira (615, 1230, or 2460 mg/kg/day) that were challenged with 50 and 100 μg of chicken egg OVA. IgG (E.U), IgM (E.U), and IgG 2a Ab (E.U) levels were detected using the ELISA Quatitation Set. Significant increases in IgG, IgM, and IgG 2a Ab levels were observed at 8 weeks in the Arthrospira 2460 mg/kg/day group (*** *p* < 0.001, compared to control). (**F**,**G**) Arthrospira enhances natural killer (NK) cell and macrophage activities in Balb/c mice. The NK cell and macrophages activities from Balb/c mice with three different doses of Arthrospira (615, 1230, or 2460 mg/kg/day) were all higher than the control group (*** *p* < 0.001, compared to control). (**H**,**I**) Arthrospira enhances IL-2 and IFN-γ levels in Balb/c mice. For innate response, the splenocytes were isolated from Balb/c mice with three different doses of Arthrospira (615, 1230, or 2460 mg/kg/day). After 5 μg/mL ConA stimulation, significant increases in IL-2 and IFN-γ levels were observed in the splenocytes from the Arthrospira treatment groups (** *p* < 0.01 and *** *p* < 0.001, compared to control). (**J**) Arthrospira enhances IgG Ab level in Balb/c mice. Sera samples were harvested at the beginning and 3 and 6 weeks from the Balb/c mice with three different doses of Arthrospira (615, 1230, or 2460 mg/kg/day). A significant increase in IgG was observed in Arthrospira treatment groups using the ELISA Quatitation Set (*** *p* < 0.001, compared to control).

Next, we examined whether Arthrospira caused B cell activation, which could be triggered by IFN-γ. As shown in Figure 5A–E, we also observed that Arthrospira enhanced specific immune response which was induced by the challenge of chicken OVA. Among the various amounts of Arthrospira treatment, dose-dependent increases in IL-2 and IFN-γ as well as increases in different antibody subtypes such as IgG, IgG-2a, and IgM were found in Balb/c mice injected with chicken OVA (Figure 5A–E).

### 3.6. Arthrospira Has the Potential to Trigger Non-Specific Immune Response

To investigate how Arthrospira modulated innate immunity, we examined several different immune cells in this aspect. NK cells constitute 1% of the total spleen immune cells and play an important role in innate immunity. We performed functional evaluation of NK cells in Arthrospira-treated cells. We examined NK cytotoxicity in primary splenocytes in the absence or presence of Arthrospira at different concentration in vitro. Splenocytes in treatment group exhibited higher killing activity compared to the control at both effector:target ratios (Figure 5F). We also examined phagocyte activity of peripheral blood mononuclear cells (PBMCs). Arthrospira treatment at three doses (615, 1230, and 2460 mg/kg/day) significantly induced macrophage phagocytosis in blood (Figure 5G). Next, we investigated the non-specific immune response of Th1 and Th2 cells after Arthrospira treatment. We examined the key proliferative cytokine IL-2 and specific polarizing cytokines, with IFN-γ as detectors. Splenocytes from mice stimulated with ConA produced higher levels of both IL-2 and IFN-γ in the presence of Arthrospira compared to levels for control cells (Figure 5H,I).

## 4. Discussion

**Figure 6:**
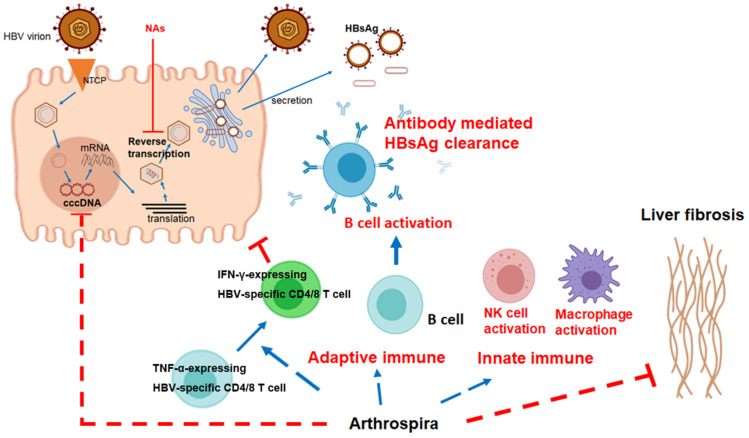
The possible mechanism of HBV reduction by Arthrospira and nucleos(t)ide analogues. First, the antiviral treatment with NAs suppresses HBV reverse transcription and DNA polymerase. Second, Arthrospira modulates the TNF-α/IFN-γ profile to trigger the differentiation of TNF-α-expressing HBV-specific CD4 and CD8 T cells into IFN-γ-expressing CD4 and CD8 T cells. Also, Arthrospira enhances the antibody-mediated reduction and clearance of HBsAg extracellularly. In addition, Arthrospira may have the potential to target the epigenetic regulation of HBV covalently closed circular DNA (cccDNA) intracellularly. Arthrospira also enhances innate immune response and attenuates HBV-induced liver fibrosis. NTCP, sodium-taurocholate co-transporting polypeptide; NAs, nucleos(t)ide analogues. Created with Biorender.com (accessed on 17 May 2022).

In this study, we explored the immune-clearance of HBV by Arthrospira supplementation. HBsAg decline is difficult to obtain. Real-world studies have shown small and slow qHBsAg declines of −0.080 to −0.098 log10 IU/mL per year in HBeAg negative patients with NA therapy [37]. Figure 2 showed a steady but small qHBsAg decline of −0.2 log_10_ IU/mL during 6 months for the standard-dose Arthrospira supplement. HBsAg < 100 IU/mL is used as a cut-off level for prediction of spontaneous HBsAg seroclearance for HBsAg loss during NA therapy [38,39]. Interestingly, the qHBsAg level decreased to below 100 IU/mL in three HBeAg-negative patients taking standard-dosage Arthrospira supplements (722 IU/mL down to 8 IU/mL; 172 IU/mL down to 29 IU/mL; 136 IU/mL down to 86 IU/mL). Our recent ongoing clinical trial (NCT04718831) showed that Arthrospira reduces HBcrAg, which reflects clearance of cccDNA (Figure 2E). In addition, the increase in serum IFN-γ parallel to the decrease in serum TNF-α levels in Arthrospira add-on groups (Figure 2F) suggested that Arthrospira stimulated the differentiation of TNF-α-expressing CD4 and CD8 T cells into IFN-γ-expressing CD4 and CD8 T cells in CHB patients. Accordingly, Arthrospira turns on the B cell adaptive immune response and the innate immune response in the Balb/c mice (Figure 5). All the data suggest that Arthrospira reduces qHBsAg through IFN-γ-B/T cell-mediated HBsAg clearance and non-specific immune response (Figure 6).

HBsAg seroclearance is set as the “ideal” end point in CHB patients under NA therapy. Although NA suppresses HBV DNA deeply, it does not affect cccDNA, which is extremely stable and a highly effective template for HBV production [40]. Therefore, it is estimated that NA therapy for several decades is required to reduce HBsAg levels, and lifelong NA treatment is necessary in almost all cases [41]. Off-NA therapy in HBeAg-negative patients with undetectable HBV DNA may increase HBsAg loss rates up to 30%/5 years [42]. Serum HBsAg may decline successively before ascending ALT reaches its peak during some hepatitis flares, followed by spontaneous resolution [42]. However, off-NA relapse with HBV flares may occur, which may result in decompensation or even death. Arthrospira enhanced HBsAg loss and cccDNA loss in CHB patients during NA treatment with mild hepatitis flares. In this study, HBsAg declined parallel to moderately increased ALT and IFN-γ expression in both HBeAg-positive and qHBsAg^low^ patients in Arthrospira add-on groups (Supplementary Figures S1C,D,G and Figure 3C,D,G). Interestingly, the ALT level (Supplementary Figure S1G) is not as high as “beneficial flare” in patients with effective qHBsAg loss rate during off-NA therapy (ALT > 5× upper limit of normal, 40 U/L) [42]. In addition, the IL-6 levels and liver stiffness degrees were decreased in Arthrospira add-on groups, suggesting that Arthrospira attenuates hepatic inflammation and fibrosis (Figure 3E,F and Supplementary Figure S1E,F). All the data indicate that the host immune response is dominating, the effective clearance of HBV is carried out by the host immune system, and the beneficial flare is limited in Arthrospira add-on groups. According to the above results, combination of Arthrospira and NA may be a safe strategy to enhance the HBsAg loss in CHB patients.

Arthrospira has the potential to trigger the differentiation of TNF-α-expressing HBV-specific CD4 and CD8 T cells into IFN-γ-expressing CD4 and CD8 T cells. HBV-specific CD4 and CD8 T cells play critical roles in viral clearance and liver injury in CHB patients. For HBV-specific CD4 T cells, TNF-α-expressing cells are the dominant population cells and play a role in liver damage, while the successful differentiation of them into IFN-γ-expressing CD4 T cells helps with HBV clearance [32]. For HBV-specific CD8 T cells, inhibition of TNF-α could enhance the cytotoxicity of IFN-γ-expressing CD8 T cells [33]. In Figure 2F, significantly increasing IFN-γ levels but decreasing TNF-α levels were observed in the Arthrospira add-on groups, suggesting that the differentiation of TNF-α-expressing CD4 and CD8 T cells into IFN-γ-expressing CD4 and CD8 T cells may be enhanced by Arthrospira. Chang’s study supported our suggestion that Phycocyanin, one of the major phycobiliproteins in Arthrospira, promoted the dendritic cell-dependent CD4 T cell stimulatory capacity and IFN-γ expression in CD4 T cells in vitro [43]. Wang’s study reported that high frequency and dominance of HBV-specific IFN-γ-expressing CD4 T cells were observed in patients with HBeAg/HBsAg loss, positively correlated with the decrease in HBsAg during flares [32]. In this study, HBsAg declined parallel to moderate ALT and IFN-γ increases in patients with HBeAg/HBsAg loss in Arthrospira add-on groups (Supplementary Figures S1C,D,G and Figure 3C,D,G), suggesting that Arthrospira may enhance the differentiation of TNF-α-expressing HBV-specific CD4 and CD8 T cells into IFN-γ-expressing CD4 and CD8 T cells, resulting in triggered HBsAg loss in CHB patients.

Arthrospira has the potential to trigger the B cell adaptive immune response in CHB patients, similarly to the interferon therapy, but is less likely to have side effects. IFN effectively boosts innate immune effectors against HBV, but numerous side effects can occur. In addition, less than 10% of HBsAg loss was reported in CHB patients with Peg-IFN-α [44]. The current standard therapy is long-term treatment with NAs such as entecavir, tenofovir disoproxil fumarate, or tenofovir alafenamide, even though interferon therapy (for 48 weeks) may be useful in some cases [45]. Many clinical trials are being carried out at this moment and promising results are anticipated, but the potential side effects of the above therapies must be carefully considered.

In the field of anti-HBV therapy, previous works have heavily researched humoral immunity, including B cell and antibody responses. Humoral immunity has attracted increasing attention again in recent years [46]. In this study, Arthrospira not only turns on the B cell adaptive immune response but also triggers the innate immune response, such as NK cell and macrophage activities (Figure 5). Another study also demonstrated the possibility of Arthrospira enhancing immunity of monocyte/macrophages. The increase of macrophage phagocytosis activities, part of non-specific immune response, in chickens due to the feeding of Arthrospira [47] is consistent with the results we found in mice (Figure 5). Arthrospira could be useful as a natural antivirus nutritional supplement. Extracts of Arthrospira have been reported to reduce the virus load as well as to improve liver function, health-related quality of life, and sexual function in patients with chronic HCV [17]. A study conducted by Chen et al. showed that Arthrospira inhibited influenza virus infection by disrupting hemagglutination [48]. A combination of Arthrospira and NAs may be a promising regimen to facilitate qHBsAg clearance in CHB patients.

The dysfunction of innate and adaptive immune response involving macrophages, NK cells, and T cells by chronic HBV persistent infection has been reported in many papers [49]. Therein, Arthrospira enhances B and T cell activation and the IL-2 and IFN-γ immune response against HBV. No patients in the add-on groups developed serious side effects. Moreover, Arthrospira has the potential to enhance HBsAg seroclearance in CHB patients via triggering the differentiation of TNF-α-expressing HBV-specific CD4 and CD8 T cells into IFN-γ-expressing CD4 and CD8 T cells, and to enhance NK cell and macrophage function. Arthrospira has the potential to not only restore immune tolerance but also reduce the risk of the hepatic carcinoma development due to decreasing the IL-6 level, liver stiffness, and hepatic steatosis in CHB patients.

There are still some limitations of our study. First, the sample size was small and the individual discrepancy was large in liver stiffness, qHBsAg, and especially IL-6 levels. To evaluate such variant outcomes, we presented relative values instead of absolute count. Second, for the immunological studies on chronic hepatitis B, HBV transgenic or hydrodymanic injected mice are much better than normal BALB/c mice. Third, the peripheral NK cell and B cell function should be evaluated to convince the immunologic effect of Arthrospira on hepatitis B virus. Finally, short-term intervention does not reflect long-term outcome of qHBsAg seroclearance and liver stiffness. For the supplementation of functional food Arthospira for patients with chronic hepatitis B, future studies should enroll more patients with long-term follow up.

## 5. Conclusions

In conclusion, Arthrospira-induced HBsAg reduction and cccDNA clearance is a new therapeutic strategy in CHB patients under long-term NA treatment (Figure 6). Immune tolerance in CHB patients can be rejuvenated by Arthrospira induced-B, T, NK, and macrophage cell activation.

## Figures and Tables

**Table 1:** Patient demographic and baseline characteristics.

Variables	Control (n = 20)	Low Dose (n = 20)	Standard Dose (n = 20)	*p* Value
**Age (years)**	49.5 ± 2.7	49.8 ± 2.3	53.8 ± 2.4	0.153
**Male**	15 (75%)	13 (65%)	15 (75%)	0.72
**Female**	5 (25%)	7 (35%)	5 (25%)	
**BMI (kg/m^2^) ^a^**	25.7 ± 5.1	26.3 ± 3.9	25.0 ± 3.8	0.646
**AST(IU/L) ^b^**	27 ± 3.3	28 ± 3.2	26 ± 3.1	0.839
**ALT (IU/L) ^c^**	28 ± 2.9	30 ± 4.1	31 ± 5.4	0.911
**Cr (mg/dL) ^d^**	0.8 ± 0.2	0.8 ± 0.2	0.9 ± 0.2	0.073
**UA (mg/dL) ^e^**	6.0 ± 1.6	5.6 ± 1.3	5.9 ± 1.4	0.667
**B12 (pg/mL)**	404.4 ± 61.7	436.8 ± 49.4	476.7 ± 43.7	0.181
**qHBsAg log_10_ (IU/mL) ^f^**	2.66 ± 0.13	2.82 ± 0.10	2.81 ± 0.15	0.618
**Fibrosis (kPa)**	7.7 ± 0.9	8.2 ± 0.9	8.1 ± 0.7	0.887

All the data are expressed as mean ± SEM. ^a^ body mass index; ^b^ aspartate aminotransferase; ^c^ alanine aminotransferase; ^d^ creatinine; ^e^ uric acid; ^f^ quantitative hepatitis B surface antigen.

## Data Availability

Data are contained within the article.

## Supplementary Materials

The following supporting information can be downloaded at https://www.mdpi.com/article/10.3390/nu14142790/s1. Figure S1: Arthrospira reduces serum qHBsAg as well as enhances serum IFN-γ level in HBeAg(+) patients receiving NA treatment; Figure S2: Arthrospira reduces serum qHBsAg and liver inflammation, as well as enhancing serum IFN-γ level in HBeAg(−) patients receiving NA treatment.
