# Supplementary material for: Arthrospira Enhances Seroclearance in Patients with Chronic Hepatitis B Receiving Nucleos(t)ide Analogue through Modulation of TNF-α/IFN-γ Profile
Source: Nutrients. 2022 Jul 6;14(14):2790. doi: 10.3390/nu14142790 (PMC9325115; doi:10.3390/nu14142790)
Supplement: Supplementary file 1 [file nutrients-14-02790-s001.zip › nutrients-1733176-supplementary.pdf]

Supplementary materials

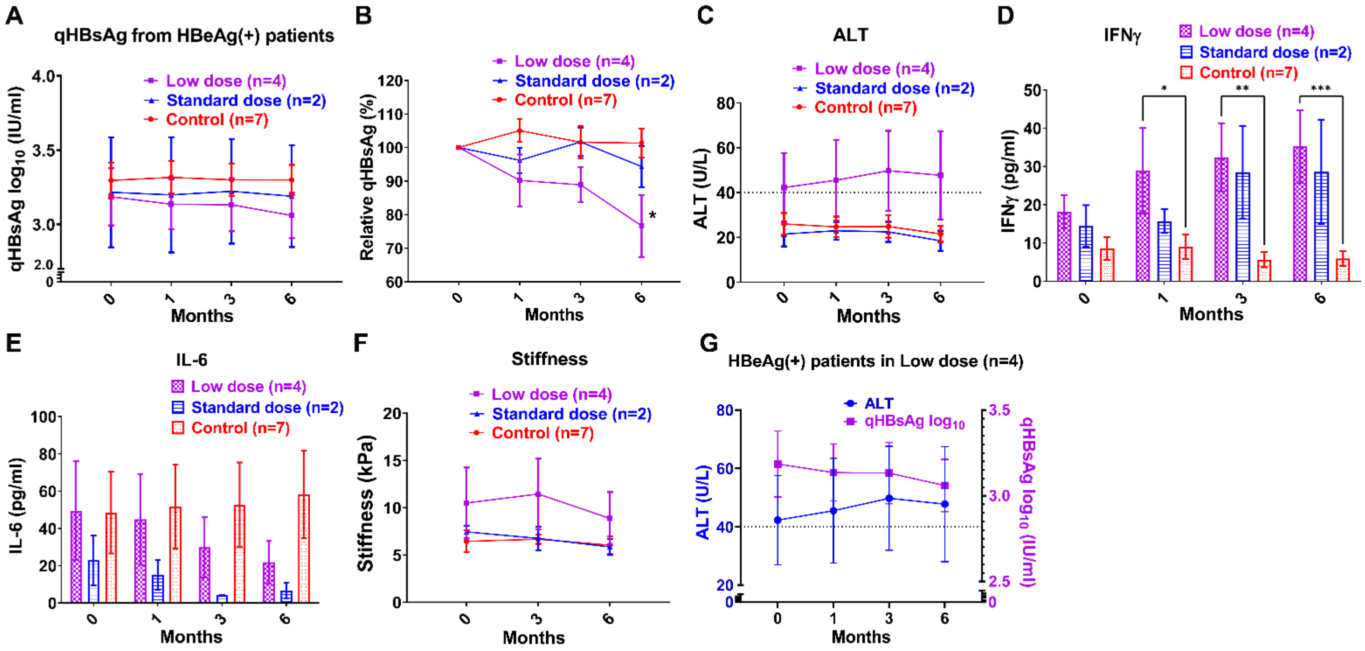

Figure S1. Arthrospira reduces serum qHBsAg as well as enhances serum IFN-γ level in HBeAg(+) patients with NA treatment. (A) The graph demonstrated the change of serum qHBsAg $\log_{10}$ (IU/ml) in patients in three groups for 6 months. (B) The graph demonstrated the change from baseline of qHBsAg in patients in three groups for 6 months. The qHBsAg level at 0 month (before Arthrospira supplement) was as the baseline level of each patient in graph B. The reduction of qHBsAg and significant reduction of relative qHBsAg level were observed in low dose group (*$p<0.05$, compared to control). (C) The ALT level was increased more than upper limit of normal, 40 U/L in low does group. (D)-(F) Serum IFN-γ level was increased but serum IL-6 level and liver stiffness degree were decreased in both Arthrospira add-on groups. Significant increase of IFN-γ level was observed at 1th, 3th and 6th month in low dose group (*$p<0.5$, **$p<0.01$ and ***$p<0.001$, compared to control). (G) Combined HBsAg/ALT kinetics in HBeAg-postive patients in low dose group (n=4). The control group: NA only, n=7; low dose group: Arthrospira 3 g daily with NA, n=4; standard dose group: Arthrospira 6 g daily with NA, n=2. Each spot represents mean ± SEM (%). The control group: NA only; low dose group: orally Arthrospira 3 g daily with NA; standard dose group: orally Arthrospira 6 g daily with NA. Each spot represents mean ± SEM (%).

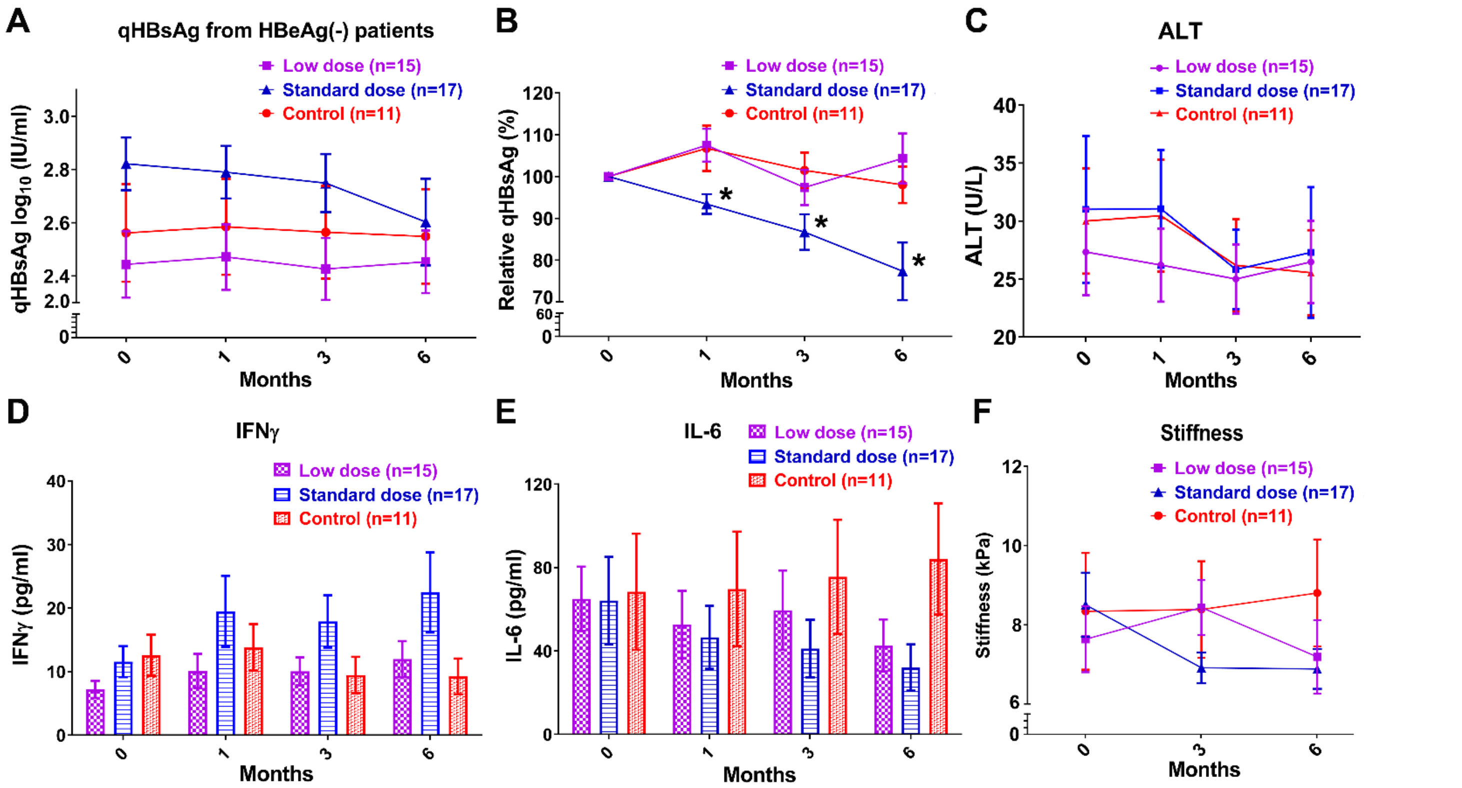

Figure S2. Arthrospira reduces serum qHBsAg and liver inflammation, as well as enhances serum IFN-γ level in HBeAg(-) patients with NA treatment.. (A) The graph demonstrated the change of serum qHBsAg log_{10} (IU/ml) in patients in three groups for 6 months. (B) The graph demonstrated the change from baseline of qHBsAg in patients in three groups for 6 months. The qHBsAg level at 0 month (before Arthrospira supplement) was as the baseline level of each patient in graph B. The reduction of qHBsAg and significant reduction of relative qHBsAg level were observed in standard dose group (*$p<0.05$, compared to control). (C) The ALT level was decreased in standard dose and control groups. (D)-(F) Serum IFN-γ level was increased but serum IL-6 level and liver stiffness degree were decreased in both Arthrospira add-on groups. The control group: NA only, n=11; low dose group: Arthrospira 3 g daily with NA, n=15; standard dose group: Arthrospira 6 g daily with NA, n=17. Each spot represents mean ± SEM (%).
